# Impact of Atrial Fibrillation Type on Quality of Life and Clinical Parameters in Patients with Diabetes Mellitus

**DOI:** 10.3390/jcdd12120453

**Published:** 2025-11-21

**Authors:** Paul Gabriel Ciubotaru, Nilima Rajpal Kundnani, Lucretia Marin-Bancila, Daniel-Dumitru Nisulescu, Nicolae Albulescu, Abhinav Sharma, Vlad-Sabin Ivan, Roxana Buzas, Veronica Ciocan, Daniel Florin Lighezan

**Affiliations:** 1 Department V, Internal Medicine I—Discipline of Medical Semiology I, Center of Advanced Research in Cardiology and Hemostasology, “Victor Babes” University of Medicine and Pharmacy, Eftimie Murgu Sq. No. 2, 300041 Timisoara, Romaniaivan.vlad@umft.ro (V.-S.I.); dlighezan@umft.ro (D.F.L.); 2 Prevention and Cardiovascular Recovery, Department VI-Cardiology, University Clinic of Internal Medicine and Ambulatory Care, “Victor Babes” University of Medicine and Pharmacy, 300041 Timisoara, Romania; knilima@umft.ro (N.R.K.); sharma.abhinav@umft.ro (A.S.); 3 Research Centre of Timisoara, Institute of Cardiovascular Diseases, “Victor Babes” University of Medicine and Pharmacy, 300041 Timisoara, Romania; 4 General Medicine Faculty, “Victor Babes” University of Medicine and Pharmacy, Eftimie Murgu Sq. No. 2, 300041 Timisoara, Romaniadaniel.nisulescu@umft.ro (D.-D.N.); 5 Division of Internal Medicine II—Cardiology, “Victor Babes” University of Medicine and Pharmacy, 3000041 Timisoara, Romania; 6 Centre for Molecular Research in Nephrology and Vascular Disease, “Victor Babes” University of Medicine and Pharmacy, 300041 Timisoara, Romania; 7 “Pius Brînzeu” Emergency County Hospital, 300723 Timisoara, Romania; 8 Department of Forensic Medicine, Bioethics, Medical Ethics and Medical Law, Center for Ethics in Human Genetic Identifications, “Victor Babes” University of Medicine and Pharmacy, 300041 Timisoara, Romania

**Keywords:** KCCQ score, glomerular filtration rate, neutrophil–lymphocyte ratio, heart failure

## Abstract

Background: Atrial fibrillation (AF) is a prevalent condition with a major influence on patients’ quality of life, especially when blood glucose and heart rate are disrupted and systemic inflammation is present. Objective: This study aimed to compare Kansas City Cardiomyopathy Questionnaire (KCCQ) scores of diabetic patients by AF type and their correlations with different clinical and biological parameters. Material and methods: The retrospective study included 220 patients, from which only 200 were selected because of missing data. Patients were divided into three groups: paroxysmal AF (*n* = 49), persistent AF (*n* = 54), and permanent AF (*n* = 97). Demographic, clinical, and analytical data, echocardiographic parameters, heart rate, blood glucose, renal function, and inflammatory markers were compared between the three groups and their relationship with KCCQ scores. Results: The KCCQ score was significantly higher in patients with paroxysmal AF (69.50 ± 5.93), compared to persistent AF (56.92 ± 3.04) and permanent AF (42.28 ± 5.89), *p* < 0.001. In subanalyses, based on left ventricular ejection fraction (LVEF), the same trend was maintained, with lower KCCQ scores associated with more severe forms of AF. Significant negative correlations of the KCCQ score with blood glucose level (r = −0.2535, *p* = 0.0003), heart rate (r = −0.3071, *p* < 0.0001), and neutrophil–lymphocyte ratio (NLR) (r = −0.2395, *p* = 0.0006), and a positive correlation with glomerular filtration rate (GFR) (r = 0.4349, *p* < 0.0001) were identified. Conclusions: The type of atrial fibrillation significantly influences the quality of life assessed by the KCCQ score. Clinical and analytical parameters such as blood glucose, heart rate, systemic inflammation, and renal function significantly correlate with patients’ perception of health, indicating the importance of integrated management of AF.

## 1. Introduction

**Figure 1:**
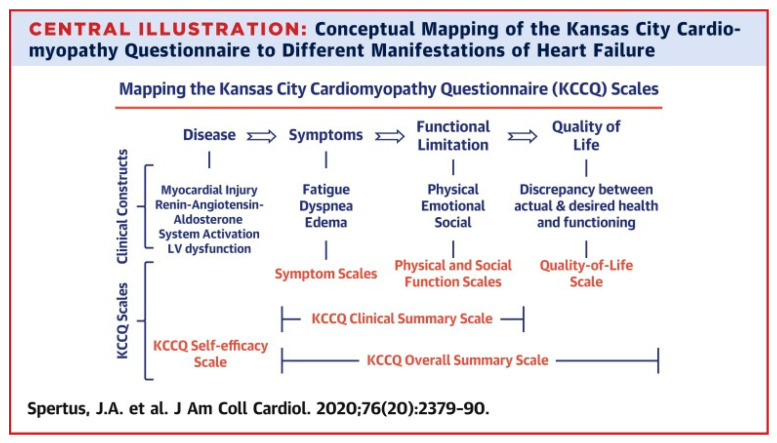
Conceptual Mapping of the KCCQ [2].

Developed in 1996 and published in 2000 [1], the KCCQ was designed with input from patients and clinicians to capture the domain of how heart failure affects patients’ lives. The KCCQ has a 2-week recall period (given the day-to-day variability in heart failure symptoms) and includes 23 items that map to 7 domains (Figure 1): symptom frequency; symptom burden; symptom stability; physical limitations; social limitations; quality of life; and self-efficacy (the patient’s understanding of how to manage their heart failure). The KCCQ was designed to capture each domain in a simple manner that was comparably assessed in men and women, older and younger patients, and across the range of patients’ socioeconomic status. To facilitate interpretation, all scores are represented on a 0-to-100-point scale, where lower scores represent more severe symptoms and/or limitations and scores of 100 indicate no symptoms, no limitations, and excellent quality of life [2].

The KCCQ was scaled down from its original 23 items (KCCQ-23) to a 12-item instrument (KCCQ-12) to make it easier to use in routine clinical care. The KCCQ-12 includes the domains of symptom frequency, physical limitations, social limitations, and quality of life. It can also produce clinical and overall summary scores that are in excellent agreement with the corresponding scores of the full instrument [3].

Atrial fibrillation (AF) is considered to be the most common clinically significant cardiac rhythm disorder and a 21st-century cardiovascular disease epidemic, having a rise in global prevalence between 2010 and 2019 from 33.5 million to 59 million individuals [4,5].

AF is heterogeneous in presentation, ranging from paroxysmal episodes to persistent or permanent forms, each associated with varying symptom burden and clinical outcomes [6,7,8]. Beyond traditional endpoints such as hospitalization and mortality, AF significantly impairs patients’ quality of life, with symptoms including palpitations, fatigue, and exercise intolerance affecting daily functioning [9]. AF is associated with increased morbidity and mortality, resulting in a high burden on the healthcare system [10]. Recognizing risk factors and intervening promptly upon their occurrence can delay the onset of AF or improve the prognosis of established AF [11]. Timely detection of AF coupled with appropriate intervention holds the potential to curtail AF-associated complications [12].

Despite being well-validated in heart failure [12], there is still little data on patient-reported outcomes in atrial fibrillation, especially when it comes to the impact of AF type, left ventricular function, and clinical or laboratory markers such as heart rate, glycemia, inflammation per se or as a marker of endothelial dysfunction [13], and renal function. Comparing KCCQ scores across AF types in diabetic patients and investigating their relationships to pertinent clinical and biochemical factors were the goals of this study.

## 2. Material and Methods

For this study, we obtained ethics approval from the Ethics Committee of the Municipal Emergency Hospital Timișoara. The study included patients who were diagnosed with atrial fibrillation and diabetes mellitus. Apart from this, the inclusion criteria comprised a completed questionnaire in the patients’ medical records and availability of all variables required for evaluation in our study. Patients with incomplete records were excluded from recruitment. Patients were divided into three groups, depending on the type of AF: paroxysmal AF (*n* = 49), persistent AF (*n* = 54), and permanent AF (*n* = 97). Paroxysmal, persistent, and permanent AF were defined according to the 2020 ESC guidelines. Paroxysmal AF was considered as self-terminating episodes lasting ≤7 days, persistent AF as episodes >7 days or requiring termination (e.g., cardioversion), and permanent AF as continuous AF accepted as permanent. AF type for each patient was assigned based on documented clinical diagnoses and ECG monitoring records in the hospital chart.

### 2.1. Data Collection

Demographic data, clinical data (systolic and diastolic blood pressure, body mass index—BMI, oxygen saturation—SaO_2_, heart rate—HR), personal pathological history (hypertension—HTN, heart failure—HF, hypercholesterolemia, type 2 diabetes mellitus, hyperuricemia, right and left bundle branch blocks—RBBB and LBBB), biochemical parameters (hemoglobin, leukocytes, neutrophils, lymphocytes, monocytes, platelets, urea, creatinine, glomerular filtration rate—GFR, blood glucose, CKMB, transaminases—ALAT and AST, alkaline phosphatase—ALAT, uric acid, D-dimers, urinary albumin and albumin/creatinine ratio—RAC) were collected, as well as echocardiographic data (atrial volume, left ventricular ejection fraction—LVEF, left ventricular hypertrophy—LVH). For grading left-sided heart failure, we followed the recommendations of the 2021 edition of the European Guidelines for the diagnosis and treatment of acute and chronic heart failure [14]. Renal function was assessed according to the 2024 KDIGO guideline for the management of chronic kidney disease [15]. Heart rate was recorded at five predefined time points—Day 1 (baseline at admission) and Days 2–5 (once daily during hospitalization under comparable resting conditions); unless otherwise specified, all other clinical and laboratory variables refer to Day 1 baseline values. Heart Rate values at D1–D5 were compared between AF groups at each time point using the same between-group framework.

### 2.2. Statistical Methods

**Figure 2:**
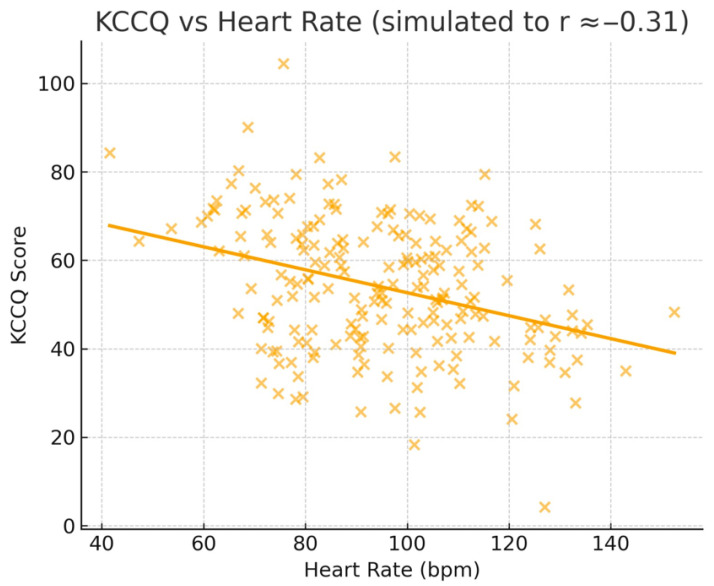
KCCQ vs. Heart Rate.

**Figure 3:**
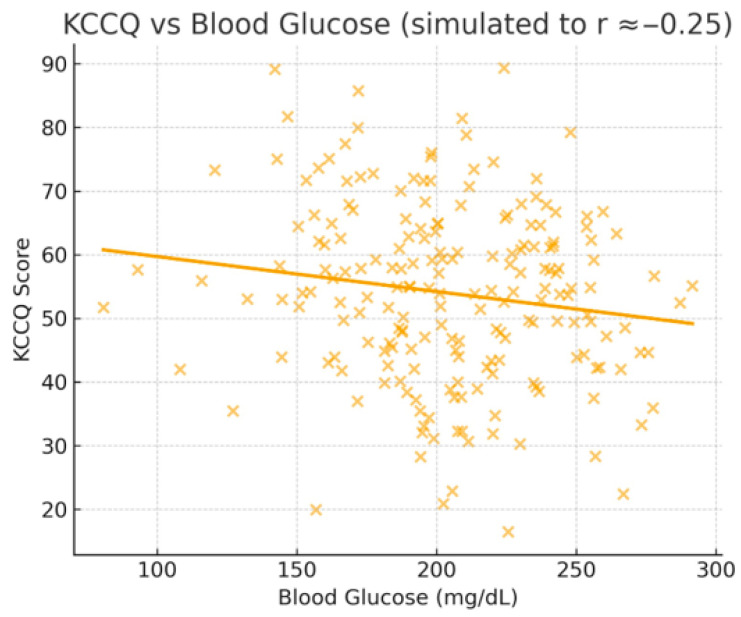
KCCQ vs. Blood glucose.

**Figure 4:**
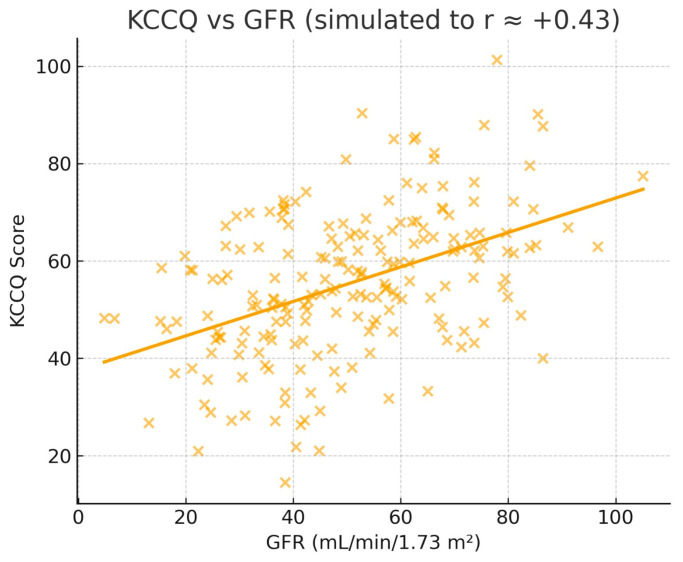
KCCQ vs. GFR.

The quality of life of the patients was assessed using the Kansas City Cardiomyopathy Questionnaire (KCCQ). Statistical analyses included ANOVA tests for comparisons between groups and Pearson and Spearman correlations to assess the relationships between KCCQ scores and clinical and biochemical parameters, which are presented in Figure 2, Figure 3 and Figure 4.

All data were analyzed using the statistical software Medcalc. Statistical significance was established for *p*-values < 0.05.

Continuous variables were checked for normal distribution (Shapiro–Wilk test). Depending on distribution, we used one-way ANOVA for between-group comparisons (with Tukey post hoc tests) or Kruskal–Wallis tests for non-parametric data. Categorical variables were compared with the Chi-square test. We considered multivariable approaches to control for potential confounders; in particular, we explored whether AF-type differences in KCCQ persisted after adjusting for age and LVEF.

## 3. Results

The retrospective study included a total of 200 consecutive patients diagnosed with atrial fibrillation (AF), who were hospitalized between 1 January 2023 and 31 December 2024 at the Municipal Clinical Emergency Hospital of Timișoara, Romania. Out of 220 patients initially assessed for eligibility, 20 had incomplete data and were excluded, yielding a final study cohort of 200 patients.

The main characteristics of the cohorts included in the analysis are presented in Table 1 and Table 2.

Analysis of the overall data reveals a similar distribution between genders and urban environment in the three groups, without statistically significant differences. The age of the patients differed significantly between the groups, with patients with permanent atrial fibrillation being the oldest (74.79 ± 10.23 years), compared to those with paroxysmal AF (69.22 ± 8.66 years, *p* = 0.004).

Hypertension, heart failure, and type 2 diabetes mellitus have a very high prevalence across all groups and were present in all patients included in the analysis. Hypercholesterolemia does not differ significantly between groups, being present in approximately 80% of patients. Chronic ischemic heart disease (CHD, *p* = 0.0110), hyperuricemia (*p* = 0.0006), and diuretic use (furosemide, spironolactone) are significantly more common in the permanent AF group. Left bundle branch block also occurs significantly more frequently in permanent AF (17.5%, *p* = 0.0393).

Patients with permanent AF have significantly lower left ventricular ejection fraction (LVEF), with nearly half of them (46.4%) exhibiting a reduced LVEF below 40% (*p* < 0.0001). Heart rate (HR) was significantly higher in the persistent and permanent AF groups at all measurement times (*p* < 0.001).

Comparative analysis of general clinical parameters between the three groups of patients (paroxysmal AF—Group 1, persistent AF—Group 2, and permanent AF—Group 3) showed significant differences in age and heart rate (HR), but not in the other measured parameters.

Patients in the permanent AF group were the oldest (74.79 ± 10.23 years), compared to patients in the persistent AF group (73.22 ± 8.13 years) and paroxysmal AF (69.22 ± 8.66 years), the difference being statistically significant (*p* = 0.004).

Systolic blood pressure did not show significant differences between groups (*p* = 0.587), being similar in mean values between the three types of AF (Group 1: 136.22 mmHg; Group 2: 131.29 ± 24.70 mmHg; Group 3: 134.54 ± 26.40 mmHg). Diastolic blood pressure did not differ significantly between groups either (*p* = 0.757).

Body mass index was comparable between groups (Group 1: 29.33 ± 5.98; Group 2: 29.03 ± 5.71; Group 3: 28.57 ± 5.55 kg/m^2^), with no statistically significant differences (*p* = 0.730).

Peripheral oxygen saturation (SaO_2_) was slightly lower in patients with permanent AF (94.75 ± 3.01%), compared to those in Group 1 (95.75 ± 2.93%) and Group 2 (95.29 ± 3.13%), but this difference was not statistically significant (*p* = 0.156).

In contrast, heart rate was significantly different between groups at all five time points analyzed D1–D5. In contrast, heart rate differed significantly between groups at all five time points D1–D5. At the first measurement, D1/baseline, HR was significantly higher in the persistent AF (108.70 ± 25.29 bpm) and permanent AF groups (106.47 ± 20.63 bpm) compared with the paroxysmal AF group (87.71 ± 24.05 bpm; *p* < 0.001). This difference remained significant at D2 and D3 (*p* < 0.001 for both), D4 (*p* = 0.020), and D5 (*p* = 0.028). Unless otherwise stated, comparisons involving other clinical or laboratory variables refer to Day-1 baseline values.

Table 3 summarizes the heart rate (HR) trends over five consecutive point in time of the hospitalization for the three AF groups. All groups showed a gradual decline in HR over time. Patients with paroxysmal AF (Group 1) had the lowest baseline HR, which decreased modestly from 87.7 ± 24.1 bpm to 77.2 ± 10.4 bpm (*p* = 0.041). In contrast, patients with persistent (Group 2) and permanent AF (Group 3) started with higher baseline HRs (108.7 ± 25.3 bpm and 106.5 ± 20.6 bpm, respectively) and demonstrated a more pronounced decline (81.7 ± 8.8 bpm and 80.1 ± 7.4 bpm, *p* < 0.001 for both groups). These results show that all forms of AF were well managed during the hospitalization with the largest reductions observed in patients with persistent and permanent AF.

- Heart rate dynamics in the three groups of patients with AF (Table 3)

The longitudinal analysis of heart rate (HR) revealed statistically significant decreases during successive measurements (D1–D5) in all three studied groups. Thus, patients in the group with paroxysmal AF (Group 1) recorded a gradual and significant reduction in HR, starting from 87.71 ± 24.05 beats/minute (D1) to 77.18 ± 10.43 beats/minute in D5 (*p* = 0.041).

Patients with persistent AF (Group 2) presented an initially much higher heart rate (108.70 ± 25.29 beats/minute), with a marked and highly statistically significant progressive decrease to 81.70 ± 8.83 beats/minute (*p* < 0.001). Similarly, patients with permanent AF (Group 3) had an initially elevated heart rate (106.47 ± 20.63 beats/minute), which decreased significantly to 80.12 ± 7.43 beats/minute at the last measurement (*p* < 0.001).

No electrical cardioversions were performed during hospitalization for any of the patients. All patients received guideline-recommended rate control therapy (e.g., beta blockers), and any antiarrhythmic medication adjustments were minimal and applied uniformly across groups. Thus, the heart rate trends observed mainly reflect the course of AF under beta blockers and or antiarrhythmic medications.

Patients with permanent AF had significantly higher neutrophils (77.55 ± 11.05%), neutrophil–lymphocyte ratio (NLR = 6.41 ± 6.09), urea (67.52 ± 28.61 mg/dl), and serum creatinine (1.60 ± 0.37 mg/dl), compared to paroxysmal AF (*p* < 0.001). Glomerular filtration rate (GFR) was significantly lower in the persistent and permanent AF groups (approx. 41 mL/min) compared to paroxysmal AF (60.95 ± 21.66 mL/min, *p* < 0.001). Glucose levels were significantly higher in patients with permanent AF, remaining consistently elevated in all measurements (D1–D5, *p* < 0.001). Cardiac injury markers (CKMB, ALAT, ASAT) and alkaline phosphatase (ALP) were significantly increased in the permanent AF group compared to the other groups (*p* < 0.001) (Table 4).

Uric acid and atrial volume were significantly higher in the permanent AF group (*p* < 0.001).

Analysis of the evolution of blood glucose levels indicated significant decreases in all three groups during the measurements (D1–D5, *p* < 0.001 for each group). The highest initial values were observed in the permanent AF group (238.63 ± 60.60 mg/dL), followed by the persistent AF group (227.88 ± 62.14 mg/dL), and the lowest were recorded in the paroxysmal AF group (196.22 ± 70.06 mg/dL). These values progressively decreased until the last assessment, maintaining the order of values between the groups. The results emphasize the significant impact of the therapeutic intervention on the reduction in blood glucose levels in all types of atrial fibrillation studied.

- Quality of life—KCCQ score

**Figure 5:**
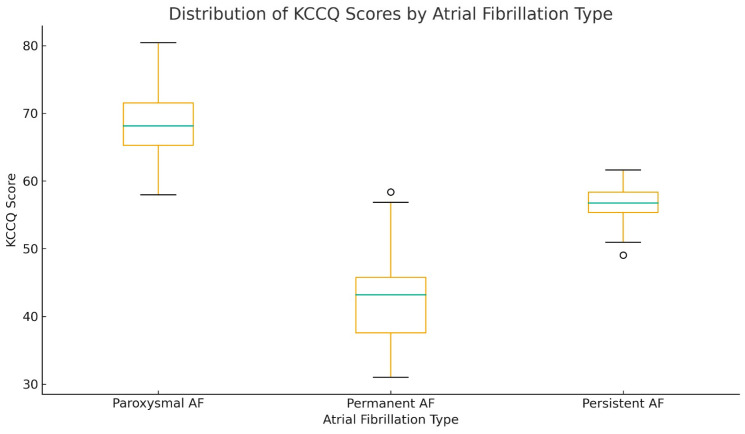
Distribution of KCCQ Scores by Atrial Fibrillation Type.

The assessment of quality of life with the Kansas City Cardiomyopathy Questionnaire (KCCQ) indicated significantly different scores between the three groups. (Table 5) The lowest score was recorded in patients with permanent AF (42.28 ± 5.89), intermediate in those with persistent AF (56.92 ± 3.04), and highest in the paroxysmal AF group (69.50 ± 5.93), with statistically significant differences (*p* < 0.001), as seen also in Figure 5. KCCQ scores were examined independently for the preserved, mid-range, and reduced EF subgroups to investigate whether left ventricular function affected patient-reported quality of life (Table 6, Table 7 and Table 8).

In the subgroup with reduced LVEF, the KCCQ score was lowest in patients with permanent AF (39.08 ± 5.67, *p* < 0.001).

In the subgroup with mid-range LVEF, KCCQ was again significantly reduced in patients with permanent (46.56 ± 3.90) and persistent (56.13 ± 4.41) AF compared to those with paroxysmal AF (70.06 ± 4.41, *p* < 0.001).

In the subgroup with preserved LVEF, the trend remained similar, with scores being significantly lower in permanent AF (43.92 ± 4.79) and persistent AF (57.29 ± 2.98) compared to paroxysmal AF (70.24 ± 6.35, *p* < 0.001).

- Correlations between KCCQ score and clinical/biochemical parameters

Pearson correlations performed on the entire group (*n* = 200) revealed significant negative relationships between KCCQ score and blood glucose also seen in Figure 2 (r = −0.2535, *p* = 0.0003), heart rate also seen in Figure 3 (r = −0.3071, *p* < 0.0001), and NLR (r = −0.2395, *p* = 0.0006), indicating that deterioration of these parameters is associated with decreased quality of life in patients with AF. A significant positive correlation was observed with GFR also seen in Figure 4 (r = 0.4349, *p* < 0.0001), highlighting the impact of renal function on patients’ perceived health status.

In the subgroup of patients with normal LVEF (*n* = 88), Spearman correlations confirmed these relationships, maintaining the same direction and significance, with emphasis on the relationships between KCCQ and blood glucose (rho = −0.220, *p* = 0.0396), heart rate (rho = −0.262, *p* = 0.0137), NLR (rho = −0.268, *p* = 0.0115) and GFR (rho = 0.355, *p* = 0.0007) (Table 9). This emphasizes that the same trends seen in the overall cohort persist even in patients with normal EF, and these clinical and biological factors are independently linked to quality of life.

## 4. Discussion

Patient-reported outcomes (PROs), which offer information on symptom burden, functional status, and general quality of life, have emerged as a crucial addition to conventional clinical measures in recent years. A popular tool for evaluating health and forecasting clinical outcomes, the Kansas City Cardiomyopathy Questionnaire (KCCQ) has undergone extensive validation in heart failure populations [16,17]. Its use in atrial fibrillation (AF) has however been limited. Prior work has suggested that AF independently reduces quality of life and increases symptom burden, as measured by disease-specific instruments or general PROs [9]. Moreover, the degree of ventricular rate control in patients with AF is directly correlated with the KCCQ score, which varies over time, including among patients with HF [18]. Our findings extend these observations by demonstrating that the KCCQ discriminates health status across AF types, correlates with comorbidities such as diabetes, and captures the impact of heart rate, systemic inflammation, and renal function. Our study results partially confirm the subanalysis of the TOPCAT and HF-ACTION trials published by Khaled et al., in which—similar to our data—the presence of AF is associated with a greater reduction in quality of life among patients with HF; however, our results contradict their claim that AF type does not influence quality of life in these patients [19]. These differences may also be explained by the longer follow-up in the studies they analyzed, as opposed to our study, which assessed changes in quality of life over a short period—one that typically preceded hospital admission for clinical deterioration. The presence of sinus rhythm in patients with heart failure is associated with a higher quality of life compared with AF in similar patients [20]. Our study likewise confirms these results: patients with HF and permanent AF had significantly lower quality of life than those with paroxysmal AF, and the characteristics of our cohort align with those typically reported for permanent AF. Also, KCCQ assesses health status factors that are relevant to AF patients, including physical limitations, overall quality of life, and symptom burden [1,2], all of which are commonly disregarded in routine clinical evaluation. Our data are “real-world”: the patients analyzed were admitted to an emergency hospital, with no exclusion criteria related to treatment regimens or mode of presentation. Analyses of quality of life outside randomized trials are less common; however, the negative impact of AF on quality of life demonstrated in our study has also been reported by published studies [21], underscoring the importance of restoring sinus rhythm in these patients.

The high prevalence of type 2 diabetes in all AF groups is a noteworthy finding in our cohort. KCCQ scores and hyperglycemia had a negative correlation, supporting earlier findings that metabolic comorbidities significantly influence symptom burden and perceived health status [17]. Similarly, renal function (GFR) exhibited a positive correlation with KCCQ scores [22], whereas heart rate and systemic inflammation (as determined by NLR) exhibited an inverse relationship. These correlations show that quality of life in AF is multifactorial and point to possible areas for integrated patient care. Direct correlations between lower GFR and poorer quality of life have been demonstrated even in patients without HF, and in these patients, inverse associations between cardiac enzyme levels and quality of life have also been reported [23]. These aspects were likewise observed in our analysis, and our study further confirms the deleterious effect of AF on patients’ quality of life.

Subgroup analyses based on left ventricular ejection fraction demonstrated that the relationship between AF type and KCCQ scores persists independently of systolic function. This result is consistent with earlier research that indicates KCCQ can distinguish between patients with varying cardiac functions, even those who are not part of traditional heart failure cohorts [24]. This emphasizes the importance of KCCQ as a complementary tool alongside conventional measures.

One important finding was that type 2 diabetes mellitus was highly prevalent in all AF groups, supporting earlier findings that diabetes is an independent risk factor for the development of AF [17,25,26]. In addition to increasing symptom burden, hospitalization rates, and mortality, diabetes is known to raise the risk of AF onset and recurrence [27]. When compared to AF patients without diabetes, patients with diabetes consistently displayed lower KCCQ scores, indicating a disproportionate impairment in health status. This result is consistent with earlier research showing that having both atrial fibrillation and type 2 diabetes substantially lowers quality of life [28,29].

An emerging point of interest is the role of sodium–glucose co-transporter 2 (SGLT2) inhibitors in AF prevention in diabetic patients. Recent studies suggest that SGLT2 inhibitors, beyond improving glycemic control and reducing heart failure admissions, may also lower the incidence of new-onset or recurrent AF [30]. The proposed mechanisms include enhanced diuresis and blood pressure control, improved left atrial remodeling and diastolic function, and anti-inflammatory effects [31]. Beyond hemodynamic, metabolic, and anti-inflammatory actions, SGLT2 inhibitors may exert direct antiarrhythmic effects by modulating atrial ionic currents. Experimental evidence summarized in a recent review indicates that SGLT2i can acutely influence key depolarizing and repolarizing currents in atrial cardiomyocytes (e.g., peak fast inward Na+ and repolarizing K+ currents), thereby stabilizing atrial electrophysiology and reducing AF vulnerability. These direct electrophysiologic actions provide a plausible mechanistic link between SGLT2i therapy and lower AF burden/recurrence [32].

These findings are supported by the correlation analyses. Significant relationships between KCCQ scores, diabetes status, and important clinical parameters like glycemia and heart rate trends were shown by both Pearson and Spearman correlations. KCCQ scores were consistently lower in patients with higher symptom burden and worse metabolic control. All of these findings suggest that diabetes not only increases the pathophysiology burden of AF, but also has a major impact on patients’ subjective perceptions of their health.

It is important to consider what the observed differences in KCCQ scores mean for patients. The KCCQ is a validated patient-reported outcome, and differences on the order of 5 points or more can reflect a noticeable change in symptom burden. In our analysis, patients with permanent AF had KCCQ scores ~27 points lower than those with paroxysmal AF, indicating a dramatically poorer quality of life. Such a gap likely corresponds to a clinically significant impact on daily functioning and possibly prognosis, as KCCQ scores have been linked to hospitalization and mortality risk in cardiovascular populations. Furthermore, the correlations suggest actionable clinical targets: for instance, patients with better heart rate control (lower resting HR) and better glycemic control tended to report higher KCCQ scores. This raises the possibility that optimizing rate control and blood glucose management could improve quality of life over time. While our study cannot prove a causal effect, it reinforces the importance of rigorous heart rate management and diabetes control in AF patients as means to potentially enhance patient-reported outcomes

Taken together, our findings suggest that KCCQs could be a patient-centered tool for managing AF, especially when it comes to monitoring high-risk subgroups like those with diabetes, elevated heart rate, systemic inflammation, or impaired renal function, as well as for assessing interventions.

Additionally, we acknowledge the potential bidirectional effects between AF and the comorbid factors we studied. For example, patients with worse renal function or heightened systemic inflammation had lower KCCQ scores in our cohort, which is intuitive since such comorbidities worsen overall health status and symptom burden. Conversely, symptomatic AF can itself contribute to hemodynamic stress and inflammatory responses. Thus, impaired renal function and inflammation may not only correlate with poorer quality of life but might also exacerbate AF severity, creating a vicious cycle. This interplay underscores the importance of a comprehensive management approach: improving underlying renal function and reducing inflammation.

It should be noted that our study did not include an AF-free control group. This was a deliberate design choice, as our objective was to compare quality of life and clinical parameters across different AF subtypes within a diabetic population, rather than to compare AF patients to those without AF. All participants had both AF and T2DM, providing a common disease context for internal comparisons. While a comparison to patients without AF, or to healthy controls, could illustrate the overall impact of AF on, that question is outside the scope of the present analysis. Instead, our findings highlight variability within AF presentations; for instance, permanent AF was associated with worse patient-reported outcomes than paroxysmal AF. We have focused our conclusions on these within-AF differences.

## 5. Study Limitations and Future Perspectives

The cross-sectional design of the study prevents any conclusions about causality, and we did not assess longitudinal outcomes such as hospitalization or mortality. Additionally, the study’s single-center setting and limited sample size restrict the generalizability of the results. Second, we did not include an AF-free or healthy control group, since the primary objective was to compare different AF subtypes within a uniform diabetic population. Consequently, the study does not quantify the absolute impact of AF versus non-AF status on quality of life, but rather focuses on within-AF variability. This was a deliberate methodological choice, allowing internal consistency by analyzing only patients who shared both atrial fibrillation and type 2 diabetes mellitus. Future studies including non-AF controls could further contextualize our findings and confirm whether the observed differences in KCCQ scores persist beyond the AF spectrum. Future multicenter studies with longer follow-up periods are needed to clarify the prognostic significance of KCCQ in AF and to determine whether interventions aimed at inflammation, glycemic control, or renal function can lead to improved patient-reported outcomes. Finally, while clinical and biological parameters were investigated, potential confounding effects from comorbidities and medications were not thoroughly considered.

## 6. Conclusions

Patients with advanced forms of AF had significantly lower KCCQ scores, which had a considerable effect on quality of life. KCCQ demonstrated a negative correlation with heart rate, glycaemia, and systemic inflammation and a positive correlation with renal function, underscoring the influence of comorbid conditions. The high prevalence of type 2 diabetes across groups further highlights its impact on health status and quality of life in AF. These findings suggest that the KCCQ may be a valuable tool for assessing patient-reported outcomes in AF, despite its initial development for heart failure.

## Figures and Tables

**Table 1:** Baseline Clinical Characteristics, Comorbidities, and Medications of the Study Cohort.

	Group 1 (*n* = 49)	Group 2 (*n* = 54)	Group 3 (*n* = 97)	*p*-Value
Gender (*n*/%)	F = 17 (34.7%) M = 32 (65.3%)	F = 26 (48.1%) M = 28 (51.9%)	F = 51 (52.6%) M = 46 (47.4%)	*p* = 0.12
Urban environment	59.2%	63%	54.6%	*p* = 0.59
HTN	100%	100%	99%	*p* = 1.0
HF	100%	100%	100%	*p* = 1.0
Hypercholesterolemia	79.6%	83.3%	84.5%	*p* = 0.75
T2DM	100%	100%	100%	*p* = 1.0
CHD chronic ischemic heart disease	24.5%	81.5%	90.7%	*p* = 0.01
Hyperuricemia	55.1%	74.1%	84.5%	*p* = 0.01
LBBB	10.2%	3.7%	17.5%	*p* = 0.03
RBBB	14.3%	11.1%	8.2%	*p* = 0.52
LVEF < 40%	22.4%	9.3%	46.4%	*p* < 0.00
LVEF 40–50%	18.4%	29.6%	23.7%	*p* = 0.40
LVEF > 50%	59.2%	61.1%	30.9%	*p* = 0.00
LVH	82.6%	88.9%	89.7%	*p* = 0.35
Beta blockers	93.9%	94.4%	94.8%	*p* = 0.97
ACEI	30.6%	37%	28.9%	*p* = 0.57
ARB	34.7%	25.9%	22.7%	*p* = 0.29
CCB	40.8%	35.2%	26.8%	*p* = 0.20
Furosemide	65.3%	87%	93.8%	*p* < 0.01
Spironolactone	57.1%	81.5%	74.2%	*p* = 0.01
DOAC	93.9%	90.7%	90.7	*p* = 0.78
Acenocumarol	6.1%	7.4%	9.3%	*p* = 0.78
Antiplatelet medication	49%	24.1%	34%	*p* = 0.02
Amiodarone	2%	18.5%	21.6%	*p* = 0.01

Abbreviations: F = female; M = male; HTN = hypertension; HF = heart failure; T2DM = type 2 diabetes mellitus; CHD = chronic ischemic heart disease (coronary heart disease); LBBB = left bundle branch block; RBBB = right bundle branch block; LVEF = left ventricular ejection fraction; LVH = left ventricular hypertrophy; ACEI = angiotensin-converting enzyme inhibitor; ARB = angiotensin II receptor blocker; CCB = calcium-channel blocker; DOAC = direct oral anticoagulant; *p* = *p*-value.

**Table 2:** Age, Blood Pressure, Oxygen Saturation and BMI Trends During Hospitalization.

	Group 1 (*n* = 49) Mean (SD)	Group 2 (*n* = 54) Mean (SD)	Group 3 (*n* = 97) Mean (SD)	*p* Value
Age (age)	69.22 (8.66)	73.22 SD 8.13	74.79 SD 10.23	*p* = 0.01
Systolic BP (mmHg)	136.22	131.29 (24.70)	134.54 (26.40)	*p* = 0.58
Diastolic BP (mmHg)	77.75 (12.91)	77.03 (13.44)	75.97 (13.44)	*p* = 0.75
BMI (Kg/m^2^)	29.33 (5.98)	29.03 (5.71)	28.57 (5.55)	*p* = 0.73
SaO_2_ (%)	95.75 (2.93)	95.29 (3.13)	94.75 (3.01)	*p* = 0.15

Abbreviations: BP = Blood pressure; SD = Standard deviation; BMI = Body mass index; SaO_2_ = Peripheral oxygen saturation (pulse oximetry); mmHg = millimeters of mercury, *p* = *p*-value.

**Table 3:** Daily Heart Rate Comparisons between the groups.

HR Mean (SD)	D1	D2	D3	D4	D5	*p* Value
Group 1	87.71 (24.05)	86.40 (19.86)	83.97(19.41)	81.51 (15.45)	77.18 (10.43)	*p* = 0.04
Group 2	108.70 (25.29)	101.18 (17.13)	95.68 (14.81)	87.57 (15.33)	81.70 (8.83)	*p* < 0.01
Group 3	106.47 (20.63)	100.43 (15.68)	94.10 (13.13)	87.36 (9.24)	80.12 (7.43)	*p* < 0.01

SD: standard deviation; HR D1–D5 = Heart rate on Day 1–Day 5.

**Table 4:** Laboratory Profiles and Between-Group Differences in AF Patients.

Mean (SD)	Group 1 (*n* = 49)	Group 2 (*n* = 54)	Group 3 (*n* = 97)	*p* Value
Neutrophils	68.77 (10.63)	74.33 (11.89)	77.55 (11.05)	*p* < 0.01
Lymphocytes	31.60 (38.51)	27.50 (41.48)	12.94 (9.76)	*p* = 0.02
NLR	3.40 (2.38)	5.11 (4.09)	6.41 (6.09)	*p* = 0.01
PLT	232.36 (58.04)	234.32 (90.60)	264.20 (96.41)	*p* = 0.045
Urea (mg/dL)	51.63 (32.01)	62.24 (25.20)	67.52 (28.61)	*p* = 0.01
Creatinine (mg/dL)	1.27 (0.45)	1.59 (0.36)	1.60 (0.37)	*p* < 0.01
GFR (mL/min/1.73 m^2^s)	60.95 (21.66)	40.98 (12.33)	40.77 (13.24)	*p* < 0.01
Serum glucose D1(mg/dL)	196.22 (70.06)	227.88 (62.13)	238.63 (60.60)	*p* = 0.01
Serum glucose D2	183.63 (54.45)	207.11 (48.15)	217.32 (54.60)	*p* = 0.01
Serum glucose D3	171.20 (45.79)	192.72 (39.13)	198.55 (41.45)	*p* = 0.01
Serum glucose D4	157.79 (33.61)	169.88 (33.40)	177.25 (33.87)	*p* = 0.01
Serum glucose D5	142.26 (27.95)	151.44 (28.98)	155.15 (32.87)	*p* = 0.01
ALAT (U/L)	30.89 (18.58)	40.85 (39.66)	76.61 (137.10)	*p* = 0.01
ASAT (U/L)	31.65 (24.13)	35.68 (28.96)	64.03 (92.59)	*p* = 0.01
ALP	72.02 (26.03)	81.87 (34.26)	101.55 (54.49)	*p* < 0.01
Albumin/creatinine ratio	140.00 (93.98)	157.96 (110.28)	177.43 (106.05)	*p* = 0.11
Urinary Albumin	60.81 (51.99)	60.18 (53.81)	76.39 (54.62)	*p* = 0.11
D dimer	2.92 (9.79)	1.66 (0.99)	4.20 (19.51)	*p* = 0.58
Atrial Volume (mL)	72.63 (24.32)	92.11 (33.34)	99.94 (35.18)	*p* < 0.01

SD: standard deviation.

**Table 5:** KCCQ scores between the three groups.

	Group 1 (*n* = 49)	Group 2 (*n* = 54)	Group 3 (*n* = 97)	*p* Value
KCCQ	69.50 (5.93)	56.92 (3.04)	42.28 (5.89)	*p* < 0.01

**Table 6:** Subanalysis KCCQ scores in patients with reduced LVEF.

	Group 1 (*n* = 11)	Group 2 (*n* = 6)	Group 3 (*n* = 45)	*p* Value
KCCQ	66.82 (5.66)	56.36 (2.77)	39.08 (5.67)	*p* < 0.01

**Table 7:** Sub-analysis: KCCQ scores in patients with mid-range LVEF.

	Group 1 (*n* = 8)	Group 2 (*n* = 16)	Group 3 (*n* = 23)	*p* Value
KCCQ	70.06 (4.41)	56.13 (4.41)	46.56 (3.90)	*p* < 0.01

**Table 8:** Sub-analysis: KCCQ scores in patients with preserved LVEF.

	Group 1 (*n* = 29)	Group 2 (*n* = 31)	Group 3 (*n* = 28)	*p* Value
KCCQ	70.24 (6.35)	57.29 (2.98)	43.92 (4.79)	*p* < 0.01

**Table 9:** Rank Correlations only patients with normal EF.

Sample Size 88	Correlation Coefficient Rho	*p* Value	95% Confidence Interval for r
KCCQ and Glycemic level	−0.220	*p* = 0.03	−0.410 to −0.0109
KCCQ and HR	−0.262	*p* = 0.01	−0.447 to −0.0556
KCCQ and NLR	−0.268	*p* = 0.01	−0.452 to −0.0625
KCCQ and GFR	0.355	*p* = 0.01	0.157 to 0.525

## Data Availability

All the data and materials will be made available on written requests.
